# Post-marketing safety of anakinra and canakinumab: a real-world pharmacovigilance study based on FDA adverse event reporting system

**DOI:** 10.3389/fphar.2025.1483669

**Published:** 2025-04-30

**Authors:** Hao Liu, Wei Yan, Jinsong Li, Dezhi Yan, Di Luo

**Affiliations:** ^1^ The First College of Clinical Medicine, Shandong University of Traditional Chinese Medicine, Jinan, Shandong, China; ^2^ Orthopedic Joint, Affiliated Hospital of Shandong University of Traditional Chinese Medicine, Jinan, Shandong, China

**Keywords:** anakinra, canakinumab, IL-1, autoinflammatory diseases, FAERS database, real-world data analysis, adverse events, pharmacovigilance

## Abstract

**Background:**

Anakinra and canakinumab are two FDA-approved IL-1 blockers indicated for the treatment of multiple autoinflammatory diseases, yet their safety has not been comprehensively analyzed. We aimed to assess the safety signals associated with anakinra and canakinumab by conducting a pharmacovigilance analysis using the FDA Adverse Event Reporting System (FAERS) database.

**Methods:**

Adverse reaction data spanning from the first quarter of 2004 to the fourth quarter of 2023 was downloaded from the FAERS database. A disproportionality analysis utilizing various methods, including the reporting odds ratio (ROR), proportional reporting ratio (PRR), Bayesian confidence propagation neural network (BCPNN), and Empirical Bayes Geometric Mean (EBGM), was conducted.

**Results:**

Anakinra and canakinumab were identified as the primary suspect drugs for adverse events (AEs) in 7,544 and 8,044 reports, respectively. The most commonly reported SOCs for both drugs were general disorders and administration site conditions. Subgroup analyses indicated that the most commonly reported SOC signals among health professionals and non-health professionals remained consistent across both medications. At the preferred term (PT) level, consistent with the drug labeling, the common AEs for anakinra and canakinumab included injection site reactions (ISRs) and infections. Further analysis revealed a higher frequency of ISRs with anakinra, including injection site pain and erythema. In contrast, canakinumab was associated with more gastrointestinal disorders (abdominal pain, mouth ulceration, and inflammatory bowel disease) and respiratory disorders (cough, oropharyngeal pain, and rhinorrhea); these conditions predominantly occurred among minors. Notably, no significant safety signals related to tuberculosis infection or reactivation were observed, and the frequency of AEs related to hepatic injury and malignancy was low.

**Conclusion:**

This study confirms the favorable safety profiles of anakinra and canakinumab, offering critical insights into rational drug usage and safety regulations.

## 1 Introduction

Interleukin 1 (IL-1), a critical proinflammatory cytokine produced by monocytes and macrophages, comprises two distinct ligands: interleukin 1α (IL-1α) and interleukin 1β (IL-1β) (Nur Sunar Yayla et al., 2024; Salmon et al., 2022). Both IL-1α and IL-1β bind to the IL-1 type 1 receptor (IL-1R1), inducing a wide range of secondary inflammatory mediators and playing critical roles in regulating immune and inflammatory processes, such as rheumatoid arthritis (RA) and systemic lupus erythematosus (Kim and Lee, 2024; Xu et al., 2024; Wu et al., 2022).

In 2001, the U.S. Food and Drug Administration (FDA) approved the first IL-1 blocker, anakinra, for the treatment of RA, cryopyrin-associated periodic syndromes (CAPS), and deficiency of IL-1 receptor antagonist (DIRA); this agent is a recombinant form of the IL-1R antagonist (IL-1Ra) that competitively inhibits the binding of IL-1α and IL-1β to the IL-1R1, thus blocking IL-1 signaling and mitigating its inflammatory effects (Arnold et al., 2022). In 2009, the FDA approved canakinumab, a human monoclonal antibody that specifically and selectively targets IL-1β, for treating periodic fever syndromes, including familial Mediterranean fever (FMF), CAPS, and Still’s disease (Sanz-Cabanillas et al., 2023). Rilonacept, another FDA-approved IL-1 blocker, was excluded from this study due to insufficient safety data. Although anakinra and canakinumab offer significant therapeutic benefits for numerous autoinflammatory diseases (AIDs), adverse drug reactions, including injection site reactions (ISRs) and infections, have been observed both during and after treatment (Giancane et al., 2022; Baverez et al., 2022; Lopalco et al., 2022; Cota-Arce et al., 2021; Qiu et al., 2024). Additionally, some studies have reported severe adverse events (AEs), such as macrophage activation syndrome (MAS), pneumonia, and liver failure (Cota-Arce et al., 2021; Kullenberg et al., 2016; Sota et al., 2018; Taylor et al., 2016). Multicenter observational studies in Europe have demonstrated that anakinra and canakinumab were frequently utilized beyond their approved indications in clinical settings, treating a spectrum of conditions from monogenic AIDs to various polygenic and multifactorial disorders, including Blau syndrome, Behçet’s disease, idiopathic uveitis, and idiopathic recurrent acute pericarditis (Vitale et al., 2016; Del Giudice et al., 2022; Rossi-Semerano et al., 2015). Significantly, this off-label use has not been accompanied by a long-term comprehensive risk assessment. Given these findings, a systematic analysis of extensive samples and real-world AEs is essential to ensure their safety.

The FDA Adverse Event Reporting System (FAERS) database, which gathers spontaneous AE reports from healthcare professionals, pharmaceutical manufacturers, patients, and others from diverse regions, is an invaluable resource for post-market surveillance and the early detection of drug safety issues (Zou et al., 2024). We conducted this pharmacovigilance study to assess the potential correlation between IL-1 blockers—anakinra and canakinumab—and AEs, utilizing real-world data from FAERS and a variety of signal quantification techniques to offer valuable insights for clinical decision-making, regulatory safety, and future comprehensive research.

## 2 Materials and methods

### 2.1 Data sources

The FAERS database has been publicly available since 2004; considering the market introduction periods of anakinra and canakinumab, this study obtained the American Standard Code for Information Interchange (ASCII) adverse event report (AER) files covering the period from the first quarter of 2004 to the fourth quarter of 2023. The categorization and standardization of AEs in the FAERS data referred to the Medical Dictionary for Regulatory Activities (MedDRA) 26.1. In the FAERS database, each report was coded with preferred terms (PTs) according to MedDRA nomenclature and categorized by system organ class (SOC) levels. Data were imported into R (version 4.4.1) for processing.

### 2.2 Data processing

Duplicate reports were removed. For data entries with the same case ID in the demographic and administrative information (DEMO) table, the most recent report based on the date was retained. Searches were conducted using the generic names “anakinra” and “canakinumab,” and trade names “kineret” and “ilaris,” respectively, to gather data on AEs reported as primary suspect (PS) drugs. These reports encompass clinical characteristics such as patient age and gender, reporter, country, and outcome. PTs of MedDRA-coded medical events with report counts ≥3 were selected, and those associated with the indications of anakinra and canakinumab were excluded. Health professionals were defined in this study as physicians, pharmacists, registered nurses, and other health professionals.

### 2.3 Disproportionality analysis

**FIGURE 1:**
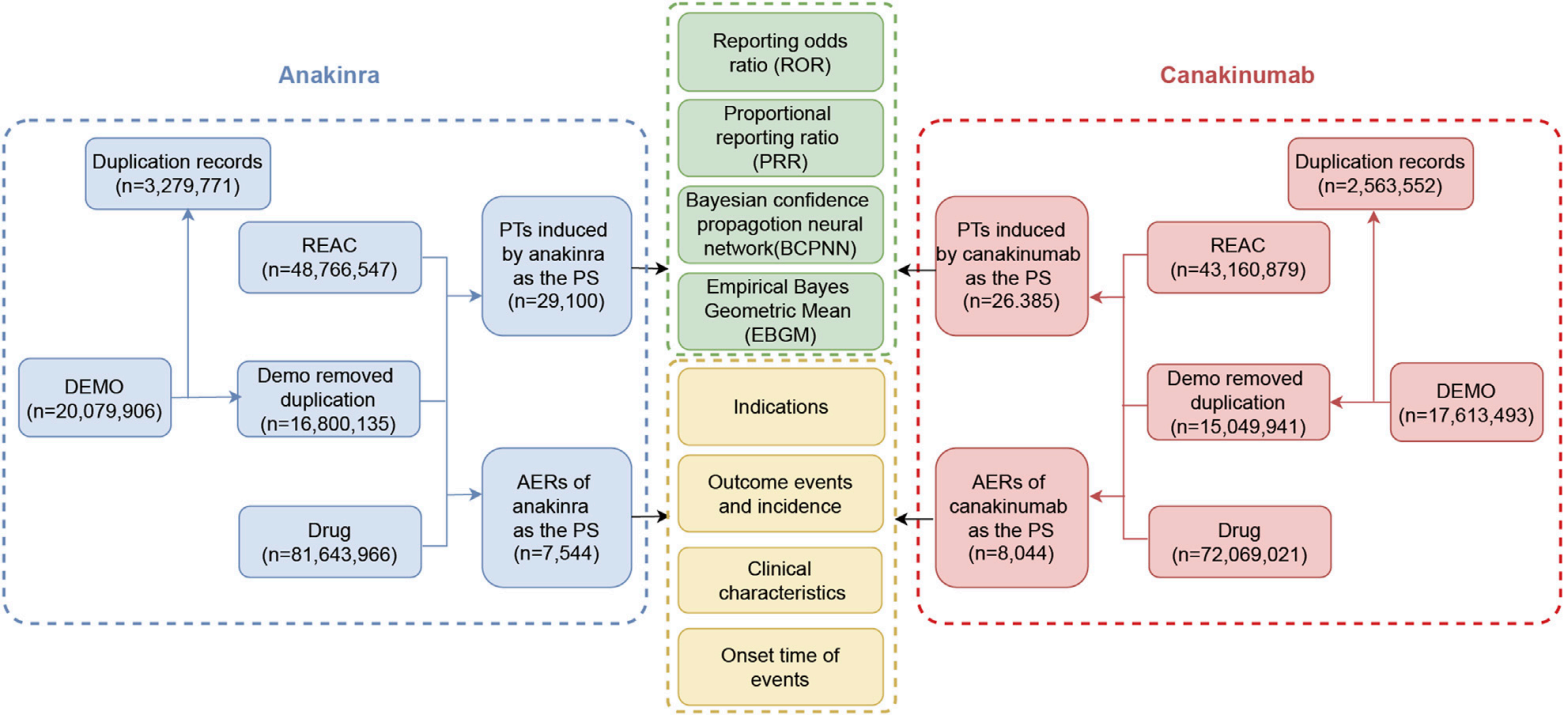
The flow diagram of the selection process for anakinra- and canakinumab-related adverse events.

Signal analysis was performed using disproportionality methods based on 2 × 2 contingency tables (Supplementary Table S1). The principle underlying these methods is to compare the frequency of an event for a target drug against the background frequency; an imbalance is noted—and thus a signal generated—when both the frequency and signal strength for the target drug and the AE exceed a specified threshold (Fusaroli et al., 2024). In this study, the reporting odds ratio (ROR), proportional reporting ratio (PRR), Bayesian confidence propagation neural network (BCPNN), and Empirical Bayes Geometric Mean (EBGM) were employed as disproportionality methods for mining AE signals. The ROR and PRR methods are noted for their high sensitivity (Moore et al., 2005); the BCPNN utilizes the Bayesian discrimination principle to enhance early-stage detection of AE signals (Tada et al., 2020), while the EBGM is adept at detecting signals of rare events (Sakaeda et al., 2013). The integrated use of these four algorithms aimed to minimize result bias inherent in single-method analyses and to detect safety signals more comprehensively and reliably. Specific formulas and threshold values are detailed in Supplementary Table S2. Statistical analysis was conducted using R software (version 4.4.1). Higher signal strength values indicate a stronger association between the target drug and the AE. The detailed data mining process is illustrated in Figure 1.

## 3 Result

### 3.1 Characteristics of adverse event reports

**FIGURE 2:**
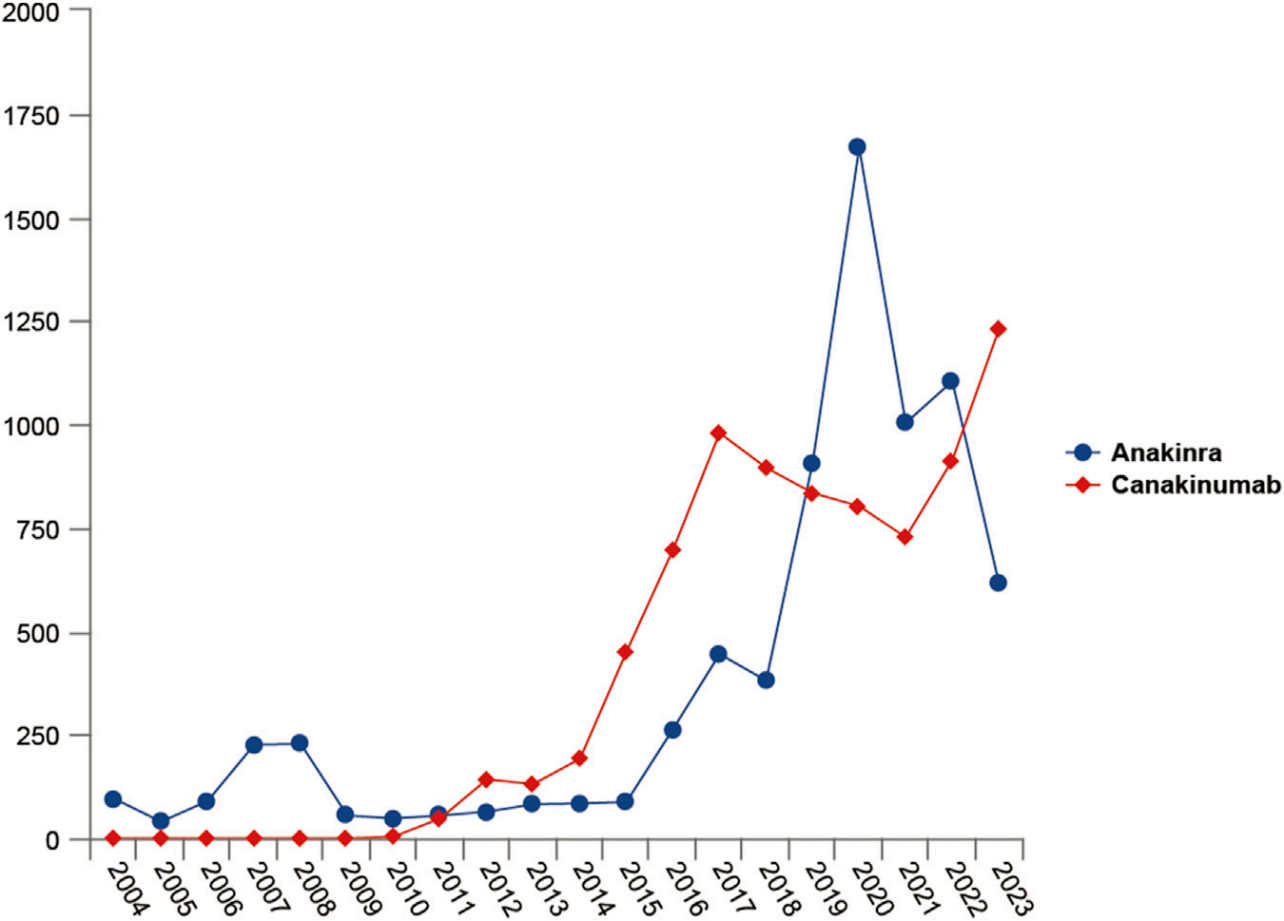
Variation of the number of adverse event reports with the year.

**FIGURE 3:**
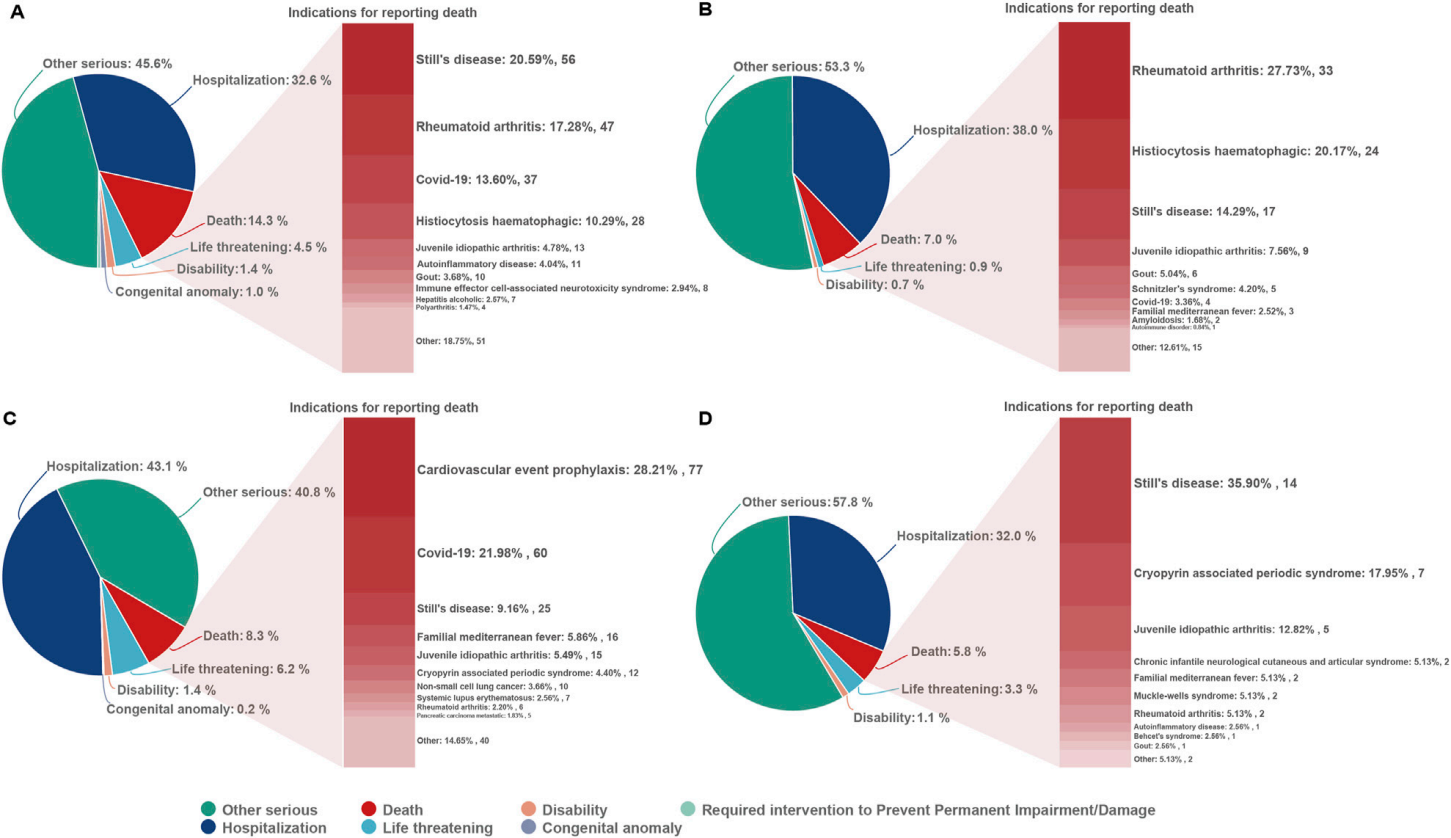
Outcomes associated with anakinra and canakinumab use, and indications associated with death reports. **(A, B)**, anakinra reported by health and non-health professionals; **(C, D)**, canakinumab reported by health and non-health professionals.

From the first quarter of 2004 through the fourth quarter of 2023, 16,800,135 AERs were obtained from the FAERS database. Of these, 7,544 and 8,044 reports identified anakinra and canakinumab, respectively, as the primary suspected drugs, documenting 29,100 and 26,385 AEs. Figure 2 illustrates the annual distribution of AERs. Table 1 depicts basic information about AERs for anakinra and canakinumab. Predominantly, the reported patients were female, accounting for 64.66% for anakinra and 53.12% for canakinumab, versus 32.90% and 37.94% male, respectively. The distribution of reports across age groups was relatively balanced for anakinra, though the 40–60 year old group had the highest incidence of AERs (14.95%), while AERs associated with canakinumab were most frequent in the <18 age group, constituting 24.96% of reports. The majority of reports were submitted by consumers (anakinra: 66.12%; canakinumab: 44.89%). The United States accounted for the highest number of reports (anakinra 71.13%; canakinumab 41.78%). The primary mode of administration was subcutaneous (anakinra: 72.64%; canakinumab: 69.24%). Of the AERs for which the time of occurrence was known, AEs associated with anakinra predominantly occurred within 30 days of dosing (32.74%), while those associated with canakinumab most frequently occurred after 540 days (17.86%). Table 2 outlines the most frequently reported indications and concomitant drugs, ranking in the top five, associated with the use of anakinra and canakinumab. Excluding unknown indications, the most prevalent conditions treated with anakinra and canakinumab were RA (26.56%) and Still’s disease (16.57%), respectively. Prednisone was the most frequently reported concomitant drug for both drugs. Regarding clinical outcomes, aside from unspecified serious AEs, hospitalization was the most frequent outcome (anakinra: 35.38%; canakinumab: 40.38%), followed by death (anakinra: 10.56%; canakinumab: 7.70%). Further analysis, as detailed in Figure 3, revealed that Still’s disease (20.59%) and cardiovascular event prophylaxis (28.21%) were the most commonly reported death-related indications among health professionals for anakinra and canakinumab, respectively.

**TABLE 1:** Basic information of adverse event reports.

Variable	Anakinra N (%)	Canakinumab N (%)
Age (years)		
<18	956 (12.67)	2,008 (24.96)
18–40	829 (10.99)	766 (9.52)
40–60	1,128 (14.95)	650 (8.08)
60–80	875 (11.60)	670 (8.33)
≥80	92 (1.22)	80 (0.99)
Unknown	3,664 (48.57)	3,870 (48.11)
Gender		
Female	4,878 (64.66)	4,273 (53.12)
Male	2,482 (32.90)	3,052 (37.94)
Unknown	184 (2.44)	719 (8.94)
Reporter		
Consumer	4,988 (66.12)	3,611 (44.89)
Physician	1,196 (15.85)	2,076 (25.81)
Pharmacist	564 (7.48)	1,524 (18.95)
Other health-professional	428 (5.67)	767 (9.54)
Unknown	358 (4.75)	63 (0.78)
Registered nurse	8 (0.11)	3 (0.04)
Lawyer	2 (0.03)	0
Country of the reports		
United States	4,741 (71.13)	3,297 (41.78)
Other	979 (14.69)	2,447 (31.01)
France	254 (3.81)	108 (1.37)
Japan	1 (0.02)	439 (5.56)
Mode of administration		
Subcutaneous	5,480 (72.64)	5,570 (69.24)
Others	1,970 (26.11)	2,350 (29.21)
Intravenous	53 (0.70)	67 (0.83)
Transplacental	41 (0.54)	14 (0.17)
Intramuscular	0	26 (0.32)
Oral	0	17 (0.21)
Outcomes		
Other serious	2,280 (49.54)	2,147 (44.94)
Hospitalization	1,628 (35.38)	1,929 (40.38)
Death	486 (10.56)	368 (7.70)
Life threatening	122 (2.65)	263 (5.51)
Disability	49 (1.06)	62 (1.30)
Congenital anomaly	24 (0.52)	7 (0.15)
Required intervention to prevent permanent impairment/damage	13 (0.28)	1 (0.02)
Time to event onset (days)		
<30	1,827 (32.74)	567 (14.33)
30–180	241 (4.32)	637 (16.09)
180–360	85 (1.52)	344 (8.69)
360–540	60 (1.08)	223 (5.63)
≥540	251 (4.50)	707 (17.86)
Unknown	3,116 (55.84)	1,480 (37.39)

**TABLE 2:** Top five indications and concomitant medications for anakinra- and canakinumab-related AEs from the FAERS database.

Variable	Anakinra (N)	Canakinumab (N)
Indications	Rheumatoid arthritis (1,731)	Product used for unknown indication (2,018)
	Still’s disease (1,008)	Still’s disease (1,294)
	Product used for unknown indication (817)	Cryopyrin-associated periodic syndrome (1,002)
	Juvenile idiopathic arthritis (540)	Pyrexia (873)
	Pericardial disease (334)	Juvenile idiopathic arthritis (798)
Concomitant Medications	Prednisone (726)	Prednisone (1,046)
	Methotrexate (415)	Colchicine (394)
	Acetaminophen (213)	Aspirin (314)
	Omeprazole (184)	Acetaminophen (285)
	Colchicine (164)	Methotrexate (205)

### 3.2 Signal of system organ class

**FIGURE 4:**
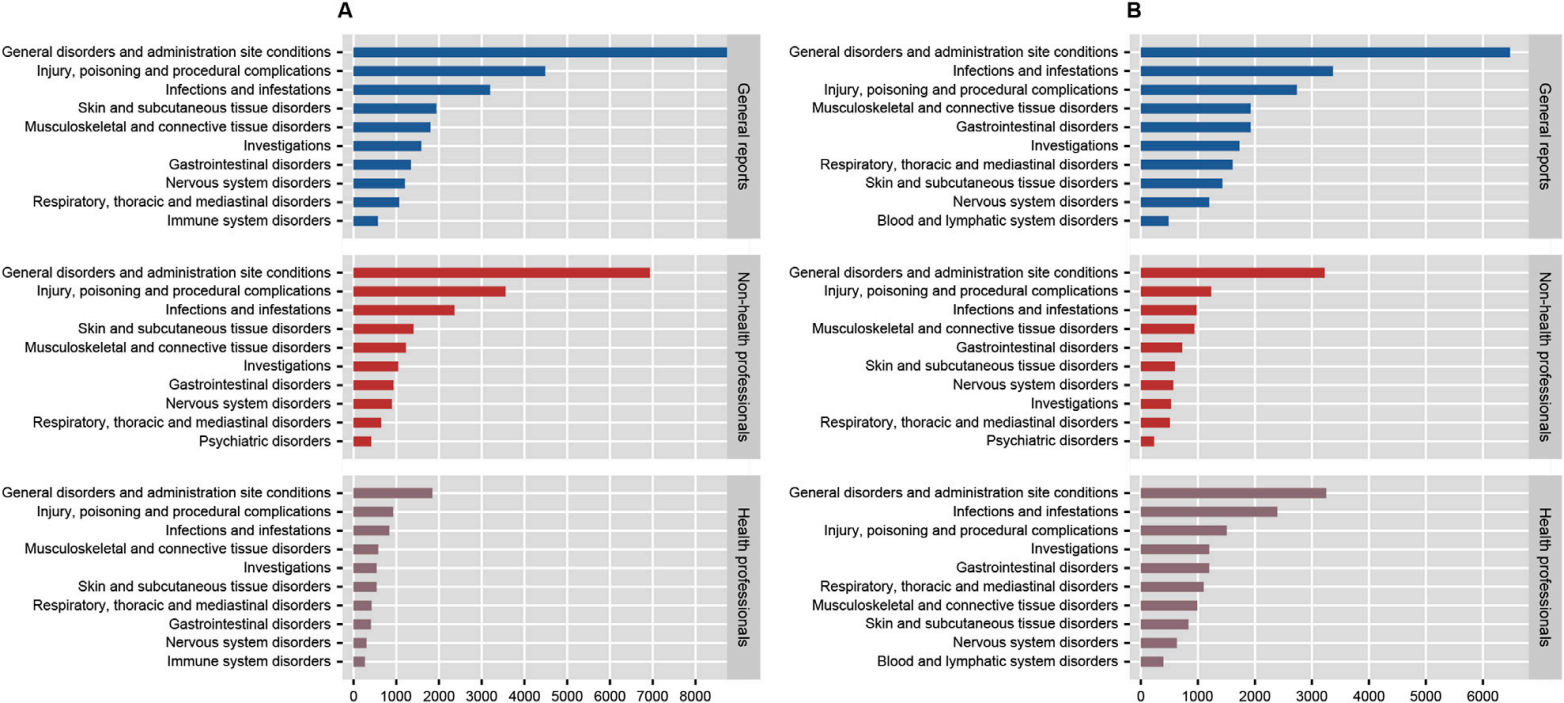
The number of system organ classes reported by health professionals, non-health professionals, and the general reported for anakinra **(A)** and canakinumab **(B)**.

In the disproportionality analysis, 24 SOCs were implicated in AEs related to both anakinra and canakinumab. The most frequently reported SOCs for anakinra included general disorders and administration site conditions (n = 8,776, ROR 1.95, PRR 1.66, IC 0.73, EBGM 1.66), injuries, poisoning and procedural complications (n = 4,483, ROR 1.76, PRR 1.64, IC 0.71, EBGM 1.64), and infections and infestations (n = 3,196, ROR 2.14, PRR 2.02, IC 1.01, EBGM 2.01). Similarly, the most reported SOCs for canakinumab encompassed general disorders and administration site conditions (n = 6,475, ROR 1.43, PRR 1.32, IC 0.40, EBGM 1.32), infections and infestations (n = 3,368, ROR 2.49, PRR 2.30, IC 1.20, EBGM 2.30), and injuries, poisoning and procedural complications (n = 2,737, ROR 1.03, PRR 1.03, IC 0.04, EBGM 1.03) (Supplementary Table S3). Additionally, comparisons of general outcomes with health professional reports indicated consistency in the most frequently reported SOCs for anakinra and canakinumab (Figure 4).

### 3.3 Signal of preferred terms

Disproportionality analysis at the PT level identified 120 significant signals for anakinra and 180 for canakinumab, including 33 identical signals (Supplementary Tables S4, S5). According to the number, the top 20 PTs are detailed in Table 3. Common PTs for anakinra included injection site pain (n = 895, ROR 6.55, PRR 6.38, IC 2.67, EBGM 6.36), injection site erythema (n = 614, ROR 10.18, PRR 9.99, IC 3.31, EBGM 9.93), and pyrexia (n = 485, ROR 2.78, PRR 2.75, IC 1.46, EBGM 2.75). Common PTs for canakinumab included pyrexia (n = 1,232, ROR 8.35, PRR 8.01, IC 3.00, EBGM 7.97), malaise (n = 669, ROR 3.18, PRR 3.12, IC 1.64, EBGM 3.12), and condition aggravated (n = 620, ROR 4.92, PRR 4.83, IC 2.27, EBGM 4.82). Table 4 details significant AE signals in death reports, noting that septic shock (n = 21) was the most prevalent AE among anakinra users, and pulmonary embolism (n = 17) among canakinumab users, as reported by health professionals.

**TABLE 3:** Significant safety signals on the PT level (top 20).

SOC	PT	Case reports	ROR (95% CI)	PRR (95% CI)	Chisq	IC (IC025)	EBGM (EBGM05)
Anakinra							
General disorders and administration site conditions	Injection site pain	895	6.55 (6.13, 7.01)	6.38 (6.02, 6.77)	4,067.63	2.67 (2.57)	6.36 (6.02)
General disorders and administration site conditions	Injection site erythema	614	10.18 (9.4, 11.03)	9.99 (9.24, 10.8)	4,947.28	3.31 (3.2)	9.93 (9.29)
General disorders and administration site conditions	Pyrexia	485	2.78 (2.54, 3.04)	2.75 (2.49, 3.03)	542.07	1.46 (1.33)	2.75 (2.55)
General disorders and administration site conditions	Injection site pruritus	470	14.56 (13.29, 15.96)	14.34 (13, 15.82)	5,791.58	3.83 (3.7)	14.23 (13.18)
General disorders and administration site conditions	Condition aggravated	409	2.92 (2.64, 3.22)	2.89 (2.62, 3.19)	506.9	1.53 (1.39)	2.89 (2.66)
General disorders and administration site conditions	Injection site reaction	399	11.84 (10.72, 13.07)	11.69 (10.6, 12.89)	3,877.24	3.54 (3.39)	11.61 (10.69)
General disorders and administration site conditions	Injection site urticaria	296	26.79 (23.87, 30.06)	26.52 (23.58, 29.83)	7,159.53	4.71 (4.54)	26.13 (23.72)
General disorders and administration site conditions	Injection site rash	287	20.66 (18.38, 23.23)	20.47 (18.2, 23.02)	5,252.54	4.34 (4.17)	20.23 (18.35)
General disorders and administration site conditions	Injection site swelling	283	7.95 (7.07, 8.94)	7.88 (7.01, 8.86)	1,693.99	2.97 (2.8)	7.85 (7.11)
Infections and infestations	Infection	274	3.97 (3.52, 4.47)	3.94 (3.5, 4.43)	601.6	1.98 (1.8)	3.93 (3.56)
General disorders and administration site conditions	Injection site bruising	272	7.28 (6.46, 8.2)	7.22 (6.42, 8.12)	1,452.51	2.85 (2.67)	7.19 (6.51)
Skin and subcutaneous tissue disorders	Urticaria	215	2.68 (2.34, 3.06)	2.67 (2.33, 3.06)	223.96	1.41 (1.22)	2.66 (2.38)
General disorders and administration site conditions	Illness	163	4.77 (4.09, 5.56)	4.75 (4.06, 5.56)	481.1	2.24 (2.02)	4.74 (4.16)
Infections and infestations	Sinusitis	155	3.02 (2.58, 3.54)	3.01 (2.57, 3.52)	207.9	1.59 (1.36)	3.01 (2.63)
Injury, poisoning and procedural complications	Contusion	146	3.04 (2.58, 3.57)	3.02 (2.58, 3.53)	197.88	1.6 (1.36)	3.02 (2.64)
Infections and infestations	Influenza	125	2.42 (2.03, 2.88)	2.41 (2.02, 2.87)	103.2	1.27 (1.02)	2.41 (2.08)
General disorders and administration site conditions	Injection site mass	124	7.01 (5.87, 8.36)	6.98 (5.85, 8.33)	633.47	2.8 (2.54)	6.96 (6)
General disorders and administration site conditions	Swelling	177	2.53 (2.11, 3.04)	2.53 (2.12, 3.02)	107.99	1.34 (1.08)	2.52 (2.17)
General disorders and administration site conditions	Injection site warmth	109	10.04 (8.31, 12.13)	10.01 (8.23, 12.18)	878.73	3.32 (3.04)	9.95 (8.5)
Infections and infestations	Cellulitis	98	3.74 (3.07, 4.56)	3.73 (3.07, 4.54)	195.46	1.9 (1.61)	3.72 (3.15)
Canakinumab							
General disorders and administration site conditions	Pyrexia	1,232	8.35 (7.89, 8.84)	8.01 (7.55, 8.5)	7,563.18	3 (2.91)	7.97 (7.6)
General disorders and administration site conditions	Malaise	669	3.18 (2.94, 3.43)	3.12 (2.88, 3.37)	972.39	1.64 (1.53)	3.12 (2.93)
General disorders and administration site conditions	Condition aggravated	620	4.92 (4.54, 5.33)	4.83 (4.47, 5.22)	1,885.01	2.27 (2.15)	4.82 (4.51)
Musculoskeletal and connective tissue disorders	Arthralgia	480	2.55 (2.33, 2.79)	2.52 (2.28, 2.78)	442.3	1.33 (1.2)	2.52 (2.33)
Skin and subcutaneous tissue disorders	Rash	434	2.27 (2.06, 2.5)	2.25 (2.04, 2.48)	302.79	1.17 (1.03)	2.25 (2.08)
Respiratory, thoracic and mediastinal disorders	Cough	289	2.27 (2.02, 2.54)	2.25 (2, 2.53)	201.81	1.17 (1)	2.25 (2.04)
Gastrointestinal disorders	Abdominal pain	235	2.28 (2.01, 2.6)	2.27 (2.02, 2.55)	167.94	1.18 (1)	2.27 (2.04)
General disorders and administration site conditions	Illness	230	6.59 (5.78, 7.5)	6.54 (5.7, 7.5)	1,076.18	2.7 (2.52)	6.52 (5.84)
Infections and infestations	Nasopharyngitis	226	2.62 (2.3, 2.99)	2.61 (2.28, 2.99)	223.95	1.38 (1.19)	2.6 (2.33)
Infections and infestations	Influenza	176	3.54 (3.05, 4.1)	3.52 (3.01, 4.12)	317.71	1.81 (1.6)	3.52 (3.11)
Infections and infestations	Infection	152	2.33 (1.99, 2.74)	2.33 (1.99, 2.73)	115.11	1.22 (0.99)	2.32 (2.03)
Respiratory, thoracic and mediastinal disorders	Oropharyngeal pain	141	3.14 (2.66, 3.71)	3.13 (2.68, 3.66)	204.52	1.64 (1.41)	3.13 (2.72)
Musculoskeletal and connective tissue disorders	Joint swelling	139	2.49 (2.11, 2.94)	2.48 (2.12, 2.9)	122.88	1.31 (1.07)	2.48 (2.16)
Respiratory, thoracic and mediastinal disorders	Rhinorrhoea	134	4.51 (3.8, 5.34)	4.49 (3.76, 5.36)	362.92	2.16 (1.92)	4.48 (3.89)
Investigations	C-reactive protein increased	131	8.57 (7.22, 10.18)	8.53 (7.15, 10.18)	867.28	3.09 (2.84)	8.49 (7.36)
Musculoskeletal and connective tissue disorders	Arthritis	119	3.21 (2.68, 3.84)	3.2 (2.68, 3.82)	179.6	1.67 (1.42)	3.19 (2.75)
General disorders and administration site conditions	Inflammation	107	4.84 (4, 5.85)	4.82 (3.96, 5.86)	323.4	2.27 (1.99)	4.81 (4.1)
Infections and infestations	Cellulitis	88	3.74 (3.03, 4.61)	3.73 (3.01, 4.63)	175.64	1.9 (1.6)	3.72 (3.13)
Respiratory, thoracic and mediastinal disorders	Nasal congestion	84	3.18 (2.57, 3.95)	3.18 (2.56, 3.95)	125.23	1.67 (1.36)	3.17 (2.65)
Blood and lymphatic system disorders	Lymphadenopathy	79	5.12 (4.1, 6.38)	5.1 (4.11, 6.33)	260.04	2.35 (2.03)	5.09 (4.23)

**TABLE 4:** Significant adverse events in death reports reported by health and non-health professionals (top 5).

Reporter	Anakinra (N)	Canakinumab (N)
Health professionals	Septic shock (21)	Pulmonary embolism (17)
	Lung disorder (12)	Acute respiratory distress syndrome (15)
	Acute respiratory distress syndrome (11)	C-reactive protein increased (12)
	Serum ferritin increased (9)	Pulmonary oedema (12)
	Drug reaction with eosinophilia and systemic symptoms (9)	Haemoglobin decreased (11)
Non-health professionals	Pyrexia (10)	Pyrexia (9)
	Condition aggravated (6)	Malaise (6)
	Pancytopenia (5)	Headache (5)
	Shock (4)	Thrombosis (3)
	Haemophagocytic lymphohistiocytosis (4)	C-reactive protein increased (3)

**FIGURE 5:**
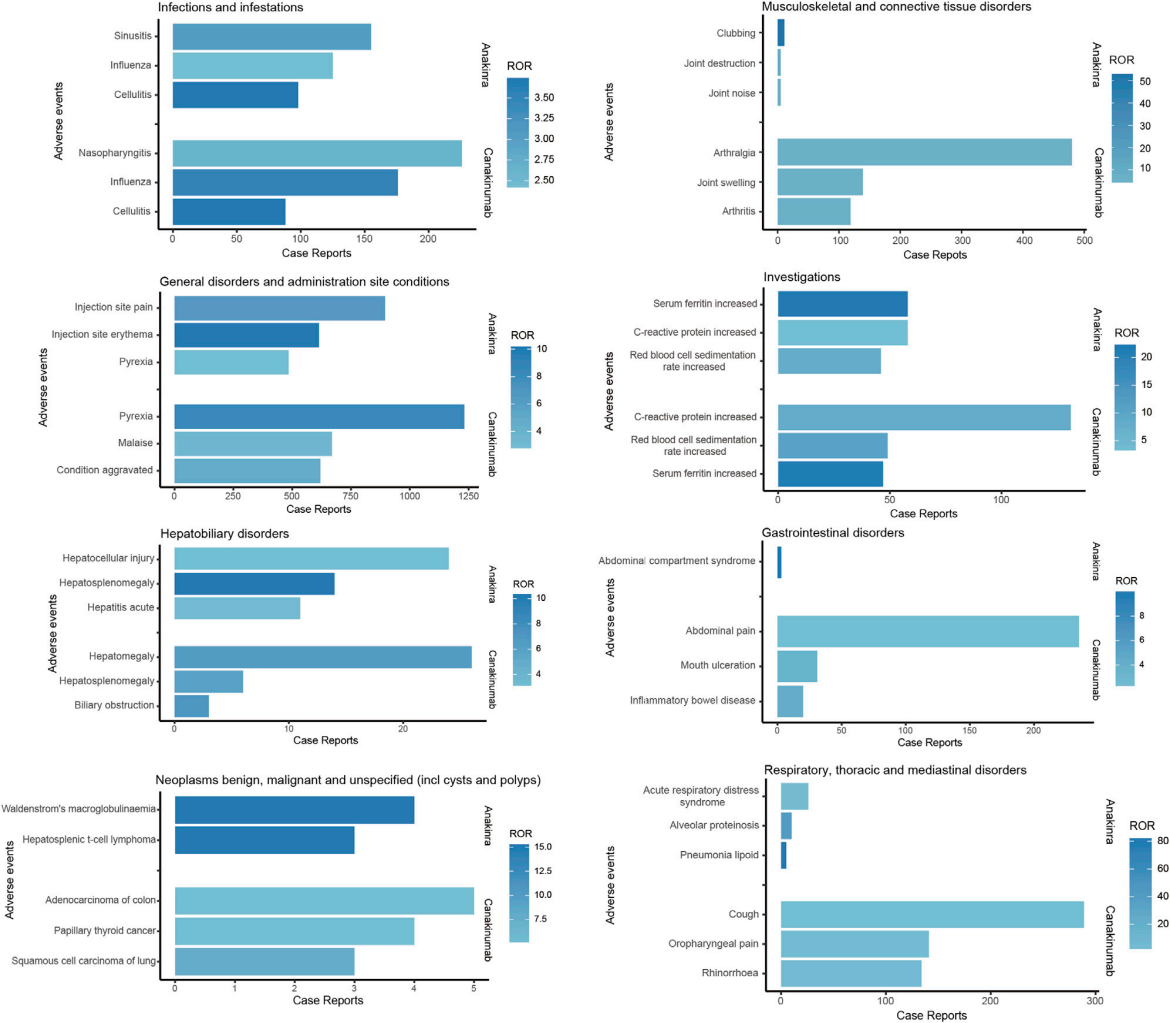
Comparison of common adverse events across various system organ classes of anakinra and canakinumab.

**FIGURE 6:**
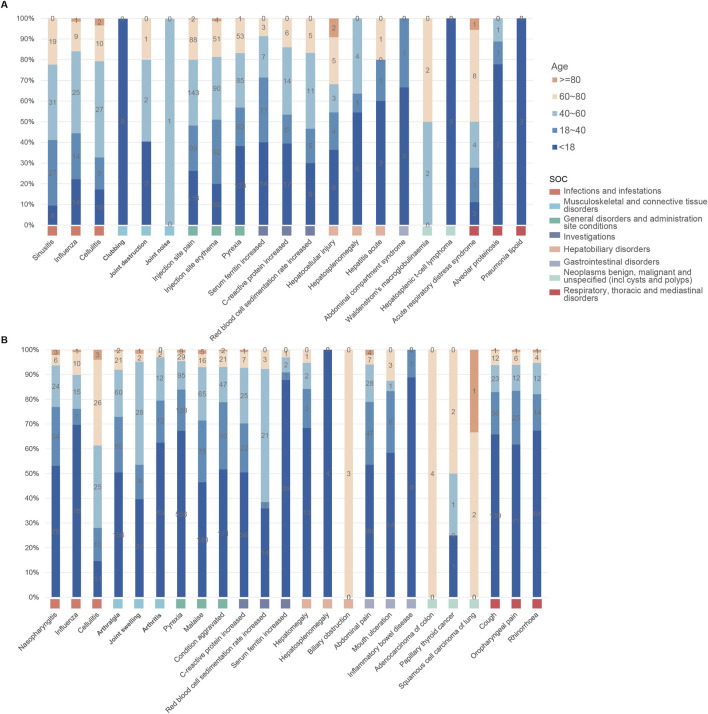
Age distribution of common adverse events across various system organ classes of anakinra **(A)** and canakinumab **(B)**.

As illustrated in Figure 5, both anakinra and canakinumab were associated with AEs such as influenza, cellulitis, and hepatosplenomegaly. Anakinra exhibited more ISRs, including injection site pain and erythema. The number of AEs was relatively evenly distributed across all age groups (Figure 6). In contrast, canakinumab was associated with a higher prevalence of abnormal investigations including C-reactive protein increased, gastrointestinal disorders including abdominal pain, mouth ulceration, and inflammatory bowel disease, and respiratory disorders including cough, oropharyngeal pain, and rhinorrhoea. The minor age group reported the highest number of AEs (Figure 6). Given the association between the immunosuppressive effects of corticosteroids (CS) and infection risk, we conducted a stratified coadministration analysis of infection- and infestation-related AEs. The analysis identified that the most significant AE signals for anakinra were necrotizing fasciitis streptococcal (with CS) and intestinal sepsis (with the other 10 most common coadministrations). In contrast, for canakinumab, they were measles (with CS) and intestinal sepsis (with the other 10 most common coadministrations) (Supplementary Tables S6, S7).

## 4 Discussion

This study represented the first systematic analysis of the safety of IL-1 blockers in a real-world population through a pharmacovigilance approach. Our analysis not only confirmed established safety information but also identified novel risk signals, thus providing a more comprehensive and accurate foundation for future public health decision-making and drug safety regulation. An in-depth discussion of the findings follows.

This study identified that subcutaneous injection emerged as the prevalent administration mode for both drugs (anakinra 72.64%; canakinumab 69.24%), followed by intravenous injection (anakinra 0.70%; canakinumab 0.83%). Although the proportion of intravenous injections was relatively low, one study demonstrated that among gout patients, intravenous administration of anakinra proved more effective than subcutaneous administration among patients with higher BMI and significant edema (Thueringer et al., 2015). Saunders et al. observed that intravenous administration of anakinra facilitated more rapid mitigation of cytokine storms in cases of adult MAS (Saunders et al., 2023). The determination of the optimal administration mode for specific indications of IL-1 blockers warrants further investigation. In addition, the significant proportion of “other” administration routes (anakinra 26.11%; canakinumab 29.21%) may obscure the risks associated with specific routes, such as intra-articular administration (Brown et al., 2011; Chevalier et al., 2009; Brown et al., 2010). Regarding the reports with established AE timing, AEs associated with anakinra most frequently occurred within 30 days of dosing (32.74%), while those for canakinumab displayed a broader temporal distribution; this discrepancy may stem from anakinra’s higher incidence of early, transient ISRs (Kaiser et al., 2012). This underscored the necessity of early and ongoing monitoring of AEs associated with anakinra and canakinumab, and indicated that future safety studies related to canakinumab may require prolonged follow-up. Regarding the countries of reporting, the majority of anakinra (71.13%) and canakinumab (41.78%) reports originated from the United States, which may not accurately reflect the global occurrence of adverse reactions, especially in regions like Asia, due to regional and racial variations. Enhanced multinational pharmacovigilance cooperation is necessary to achieve a comprehensive understanding of adverse reaction profiles worldwide.

Analysis of indications, with the exception of unknown product uses, revealed that RA (26.56%), Still’s disease (15.47%), and juvenile idiopathic arthritis (12.54%) were the prevalent indications for anakinra, while for canakinumab, common indications included Still’s disease (16.57%), CAPS (12.83%), and pyrexia (11.18%). Notably, both drugs demonstrated a significant frequency of off-label use, especially anakinra. The emerging recognition that IL-1 plays a pivotal role in the immune-driven mechanisms of inflammatory diseases, and that IL-1 blockers inhibit proinflammatory pathways in these conditions through a highly specific mode of action, has prompted the increased use of targeted modulatory IL-1 therapies across various inflammatory diseases (Sota et al., 2018). Recent systematic reviews have shown that anakinra and canakinumab both provide significant clinical benefits in treating CAPS, FMF, gout, RA, and systemic juvenile idiopathic arthritis (Arnold et al., 2022). In alignment with our findings, multicenter observational studies from Europe have revealed that, due to limited licensed indications, anakinra and canakinumab were frequently administered off-label, particularly anakinra (Vitale et al., 2016; Rossi-Semerano et al., 2015). Anakinra was employed as a first-line biologic agent in a larger patient cohort, potentially explaining the drug’s comparatively higher rate of off-label prescribing (Vitale et al., 2016). Indeed, the current experience with IL-1 blockers in off-label settings proves inadequate due to the absence of comprehensive long-term monitoring. Our exploration of AEs using real-world data offers critical evidence towards ensuring their safety.

A series of risk signals were identified in this study; based on disproportionality analysis at the SOC level, AEs associated with anakinra were primarily linked to infections and infestations, while those associated with canakinumab predominantly involved congenital, familial and genetic disorders and infections and infestations. Of note, the majority of AERs were submitted by consumers, rather than healthcare professionals. Considering the potential lack of reliability and comprehensiveness in consumer reports compared to professional submissions, as well as the differences in focus—such as patients potentially excelling at reporting subjective experiences—we subsequently analyzed a subset of reports submitted by both healthcare and non-healthcare professionals, comparing these with the overall findings. The results indicated only minor changes in the rankings of the most frequently reported SOC signals, suggesting minimal impact of reporting bias on our conclusions, further affirming the reliability of our findings.

At the PT level, both anakinra and canakinumab exhibited significant signals of AEs related to infections and ISRs. These AEs generally aligned with the information provided in the drug insert and clinical safety data. Although previous studies reported favorable safety profiles for anakinra and canakinumab (Atas et al., 2021; Arnold et al., 2022), our findings indicated that occurrences of death were documented in AER outcomes. Further analysis of these reports revealed that Still’s disease and cardioembolic event prophylaxis were the primary indications reported by health professionals for anakinra and canakinumab, respectively. AEs such as septic shock, acute respiratory distress syndrome, and condition aggravated occurred frequently, suggesting that the poorer general clinical status of the underlying disease, its complications, and further deterioration may be the principal contributors to mortality.

ISRs represent the most common and consistently reported AEs for IL-1 blockers. A retrospective observational study demonstrated that canakinumab was associated with a lower rate of ISRs compared to other IL-1 blockers (Nur Sunar Yayla et al., 2024). Consistent with these observations, we found that a greater number of ISRs was associated with anakinra, attributable to the shorter half-life of anakinra and the requirement for daily subcutaneous injections. In contrast, canakinumab, with its longer half-life, does not require frequent injections or high dosages, potentially contributing to better skin tolerance of canakinumab (Dhimolea, 2010). Furthermore, the analysis indicated that both drugs were linked to improper drug use, particularly anakinra. Anakinra is comparatively inconvenient compared with canakinumab, which requires injections every 4 or 8 weeks. The more frequent ISRs associated with anakinra may also have impeded patient compliance.

As with all biologics, infections represent a worrisome adverse effect of IL-1 blockers. Cabral et al. observed that 129 (5.1%) of 2,896 RA patients treated with anakinra developed serious infections, primarily in the respiratory tract (Cabral et al., 2016). A meta-analysis demonstrated a dose-dependent increase in the incidence of serious infections among 2,062 RA patients undergoing treatment with anakinra (Salliot et al., 2009). Furthermore, numerous observational studies have disclosed that infections, particularly upper respiratory tract infections (URTIs), are the most common AEs in canakinumab-treated pediatric rheumatic diseases (Kilic Konte et al., 2024; Coşkuner et al., 2023; Iwata et al., 2023). In alignment with prior findings, this study identified a high frequency of infection-related AEs with strong signal intensity; sinusitis and nasopharyngitis were the most frequent infection-related AEs associated with anakinra and canakinumab, respectively. Importantly, the present study did not detect a safety signal for tuberculosis infection or reactivation. Previous evidence indicated that opportunistic infections, specifically those caused by *Mycobacterium tuberculosis*, were rare in patients treated with IL-1 blockers, even among populations at high risk for reactivation of latent infections (Cavalli and Dinarello, 2018; Cantarini et al., 2015; Dumaine et al., 2020; Lima et al., 2023). IL-1 appears to be associated with late hypersensitivity reactions to *M. tuberculosis*. However, this association does not play a fundamental role in infection control, potentially explaining the minimal or absent risk of tuberculosis in patients treated with IL-1 blockers (Lima et al., 2023). Nonetheless, given the elevated risk of infection associated with IL-1 blockers, it is recommended that thorough assessments be conducted for active and latent infections before initiating treatment, and that indicators of infection are closely monitored during therapy to minimize the risk of severe infectious events.

Although hepatotoxicity associated with IL-1 blockers is rare, it cannot be ignored. Multiple case reports have documented hepatotoxicity related to anakinra use in both adult and pediatric patients, particularly in those with Still’s disease or predisposing factors, such as a history of elevated liver enzymes (Martins et al., 2023; Murray et al., 2021; Giancane et al., 2022). Taylor et al. reported a case wherein a teenager with adult-onset Still’s disease developed severe acute hepatic failure attributed to anakinra use (Taylor et al., 2016). Canakinumab exerts minimal impact on hepatic metabolism compared to anakinra and has not been linked to any clinically significant cases of acute liver injury (Gülez et al., 2020). However, multiple large registration trials have reported ALT elevations in 1%–3% of patients treated with canakinumab (Lachmann et al., 2009; So et al., 2010; Schlesinger et al., 2012; Ruperto et al., 2018; Feist et al., 2018). This study identified significant signals of hepatocellular injury and hepatomegaly in anakinra-associated AEs, as well as hepatomegaly and transaminase elevation in canakinumab-associated AEs, thus confirming the association between IL-1 blockers and liver injury. Notably, these signals predominantly occurred in minors. The absence of systematic liver function monitoring in FAERS data could result in detection bias and an underestimation of risk since mild or transient liver enzyme elevations might be undocumented. Consequently, these results must be interpreted with caution. The mechanisms underlying hepatic injury caused by anakinra and canakinumab remain elusive and are potentially linked to their effects on the immune system or the IL-1 pathway, crucial for regulating inflammation and cellular damage. Although severe hepatic injury AEs are rare, monitoring liver function during IL-1 blocker therapy is essential, particularly in minors. Further research is required to elucidate the direct connection between anakinra, canakinumab, and liver injury.

The study also identified numerous significant risk signals not outlined in the drug labels, including clubbing, Waldenström’s macroglobulinemia, hepatosplenic T-cell lymphoma, body height below normal, and decreased gastric pH associated with anakinra use; and deafness, pulmonary thrombosis, brain edema, clubbing, increased intracranial pressure, adenocarcinoma of the colon, papillary thyroid cancer, and squamous cell carcinoma of the lung related to canakinumab use. All of these AEs occurred infrequently; their mechanisms have yet to be fully elucidated, and certain side effects may be a general result of impaired IL-1 pathways. It is noteworthy that we identified rare tumor-associated AEs with both anakinra and canakinumab. A multicenter study reported Waldenström’s macroglobulinemia in two patients with Schnitzler’s syndrome, attributed to anakinra use (Néel et al., 2014). The literature still lacks a conclusive association between IL-1 blockers and malignancy; though the frequency of malignancy-associated AEs is low, prospective, long-term multicenter studies remain necessary.

## 5 Limitations

Although the comprehensive characterization of anakinra and canakinumab-associated AEs in this study provided robust evidence for their safety, several limitations persist. First, the FAERS database depends on a spontaneous reporting system, which carries inherent risks such as irregular and incomplete reporting, potentially leading to reporting bias. Second, the signal of AEs detected by the study using the disproportionality method elucidates only a statistical correlation with the targeted AEs, not representing a biological causal link; further clinical studies are therefore necessary to explore this causal relationship. Additionally, the scarcity of detailed clinical patient information and medication information hindered control over confounding variables, including comorbidities, medication dosage, and other health-affecting factors, as well as the ability to assess efficacy. Finally, given the voluntary reporting nature of the FAERS database, it is not possible to determine the total number of individuals treated and thus the exact incidence of AEs and mortality outcomes.

## 6 Conclusion

Our comprehensive pharmacovigilance analysis of the FAERS database contributes real-world evidence supporting the clinical safety management of anakinra and canakinumab. Overall, the safety profile of anakinra and canakinumab was generally favorable, characterized primarily by ISRs and infections. No significant safety signals were detected for tuberculosis infection or reactivation, and the incidence of hepatic injury and malignancy-associated AEs was low. Furthermore, we observed that both anakinra and canakinumab were frequently used beyond their approved indications. In this context, the multilevel analysis of this study serves as a crucial reference for clinicians to optimize drug selection and enhance safety regulatory efforts. Future research, including more rigorous prospective, multicenter clinical trials and epidemiological studies, is necessary to validate our findings and facilitate a more precise assessment of the safety risks associated with anakinra and canakinumab.

## Data Availability

The original contributions presented in the study are included in the article/Supplementary Material, further inquiries can be directed to the corresponding author.
